# A General Map of Transcriptional Expression of Virulence, Metabolism, and Biofilm Formation Adaptive Changes of *Staphylococcus aureus* When Exposed to Different Antimicrobials

**DOI:** 10.3389/fmicb.2022.825041

**Published:** 2022-06-17

**Authors:** Zun Ren, Jinlong Yu, Jiafei Du, Yubo Zhang, Musha Hamushan, Feng Jiang, Feiyang Zhang, Boyong Wang, Jin Tang, Hao Shen, Pei Han

**Affiliations:** ^1^Department of Orthopedics, Shanghai Jiao Tong University Affiliated Sixth People’s Hospital, Shanghai, China; ^2^Department of Clinical Laboratory, Shanghai Jiao Tong University Affiliated Sixth People’s Hospital, Shanghai, China; ^3^Department of Orthopedics, Shanghai Sixth People’s Hospital Fujian, Jinjiang, China

**Keywords:** *Staphylococcus aureus*, antimicrobials, biofilm formation, virulence, metabolism

## Abstract

Biofilm formation of *Staphylococcus aureus* is the major cause of implant-associated infections (IAIs). Antimicrobial treatment is one of the most effective therapeutic options for *S. aureus* infections. However, it can also lead to adaptive transcriptomic changes due to extreme selective pressure, which may increase the risk of antimicrobial resistance. To study the transcriptional changes in *S. aureus* upon exposure to antimicrobial agents, we obtained expression profiles of *S. aureus* treated with six antimicrobials (flucloxacillin, vancomycin, ciprofloxacin, clindamycin, erythromycin, and linezolid, *n* = 6 for each group). We also included an untreated control group (*n* = 8) downloaded from the Gene Expression Omnibus (GEO) database (GSE70043, GSE56100) for integrated bioinformatic analyses. We identified 82 (44 up, 38 down) and 53 (17 up, 36 down) differentially expressed genes (DEGs) in logarithmic and stationary phases, respectively. When exposed to different antimicrobial agents, we found that manganese import system genes and immune response gene *sbi* (immunoglobulin G-binding protein Sbi) were upregulated in *S. aureus* at all stages. During the logarithmic phase, we observed adaptive transcriptomic changes in *S. aureus* mainly in the stability of protein synthesis, adhesion, and biofilm formation. In the stationary phase, we observed a downregulation in genes related to amino biosynthesis, ATP synthesis, and DNA replication. We verified these results by qPCR. Importantly, these results could help our understanding of the molecular mechanisms underlying the proliferation and antimicrobial resistance of *S. aureus*.

## Introduction

*Staphylococcus aureus* is a gram-positive aerobic bacterium that causes various human infections (Troeman et al., 2019) including implant-associated infections (IAIs). IAIs caused by biofilm formation of *S. aureus* are still significant clinical issues, which can lead to morbidity, mortality, and increased healthcare expenditure (Klevens et al., 2007). Biofilm is a complex community of microorganisms adhered to surfaces and surrounded by protective extracellular polymeric substances (EPS) (Mirzaei et al., 2021). The biofilm formation goes through four stages comprising adherence, aggregation, maturation, and detachment. Proteinaceous and polysaccharide-dependent procedures are the two related mechanisms of biofilm formation in *S. aureus*, among which the polysaccharide intercellular adhesin (PIA) produced by the *ica* operon is the best-understood mechanism (Mirzaei et al., 2016, 2019). The biofilms are responsible for the resistance to antimicrobial agents and immune system defense.

The use of antimicrobials is one of the most effective strategies against *S. aureus* infection. Interruption of bacterial cell wall synthesis, suppression of bacterial protein synthesis, and inhibition of bacterial DNA gyrase are the major mechanisms of action for antimicrobials (Chernov et al., 2019). Although high-dose antimicrobials can eliminate bacteria, adaptive changes may occur in *S. aureus* due to the extreme selection pressure from the antimicrobial agents themselves. This can increase the risk of antimicrobial resistance and/or other pathological processes (Bui et al., 2017). In the presence of an implant, low-dose or long-term antimicrobial application can even promote biofilm formation in *S. aureus*, leading to reduced sensitivity to antimicrobial treatments (Lamret et al., 2020). This acquired antibiotic resistance is crucial for the survival of *S. aureus* (Proctor et al., 2006).

Researching the common adaptive transcriptional expression of biofilm formation, virulence, and metabolism of *S. aureus* when exposed to different low-dose antimicrobials can aid our understanding of the molecular mechanisms underlying the proliferation and induced antimicrobial resistance of *S. aureus*. This will have clinical significance for the prevention of *S. aureus*-induced IAIs and it will also aid the identification of novel targets against *S. aureus*.

To this end, we used public database resources to download gene expression profiles of GSE70043 (Mäder et al., 2016) and GSE56100 (Varma et al., 2019) from the Gene Expression Omnibus (GEO database). These expression matrixes contain the transcriptional data of *S. aureus* that have been exposed to various antimicrobials in different growth phases. After screening the differentially expressed genes (DEGs), we analyzed the biological function of the highlighted genes and verified these findings by qPCR. Herein, we propose a general map of transcriptional expression during biofilm formation, virulence, and metabolism following antimicrobials-induced adaptive changes in *S. aureus.*

## Materials and Methods

### The Information of Microarray Data and R **Packages**

The gene expression profiles of GSE70043 (Mäder et al., 2016) and GSE56100 (Varma et al., 2019) were downloaded from the GEO DataSets database,^1^ ^,2^ which were identified by searching the keywords “staphylococcus aureus antimicrobials.” The dataset information is shown in Table 1. All bioinformatic analyses were performed in R(4.0.2). The getGEO function (GEOquery package) was used to download and process files. The data were normalized by the “normalizeBetweenArrays” function (limma package; Ritchie et al., 2015).

**TABLE 1:** Details for GEO data.

GEO	Platform	Strain	Sample	Replicates	group	Treatment	Stage
GSE70043	GPL20586	NCTC 8325	Fluc-log	3	Treatment	Flucloxacillin	Logarithmic
GSE70043	GPL20586	NCTC 8325	Fluc-sta	3	Treatment	Flucloxacillin	Stationary
GSE70043	GPL20586	NCTC 8325	Vanc-log	3	Treatment	Vancomycin	Logarithmic
GSE70043	GPL20586	NCTC 8325	Vanc-sta	3	Treatment	Vancomycin	Stationary
GSE70043	GPL20586	NCTC 8325	Cipro-log	3	Treatment	Ciprofloxacin	Logarithmic
GSE70043	GPL20586	NCTC 8325	Cipro-sta	3	Treatment	Ciprofloxacin	Stationary
GSE70043	GPL20586	NCTC 8325	Clind-log	3	Treatment	Clindamycin	Logarithmic
GSE70043	GPL20586	NCTC 8325	Clind-sta	3	Treatment	Clindamycin	Stationary
GSE70043	GPL20586	NCTC 8325	Ery-log	3	Treatment	Erythromycin	Logarithmic
GSE70043	GPL20586	NCTC 8325	Ery-sta	3	Treatment	Erythromycin	Stationary
GSE70043	GPL20586	NCTC 8325	Line-log	3	Treatment	Linezolid	Logarithmic
GSE70043	GPL20586	NCTC 8325	Line-sta	3	Treatment	Linezolid	Stationary
GSE70043	GPL20586	NCTC 8325	Con-log	3	Control	TSB medium	Logarithmic
GSE70043	GPL20586	NCTC 8325	Con-sta	3	Control	TSB medium	Stationary
GSE56100	GPL18450	ATCC29213	Celecoxib	2	Treatment	Celecoxib	Stationary
GSE56100	GPL18450	ATCC29213	Con	2	Control	TSB medium	Stationary

*GEO, Gene Expression Omnibus; Fluc, flucloxacillin; Vanc, vancomycin; Cipro, ciprofloxacin; Clind, clindamycin; Ery, erythromycin; Line, linezolid; Log, logarithmic phase; Sta, stationary phase.*

### Differentially Expressed Genes **Analysis**

The platform with annotation information was downloaded using the getGEO function (GEOquery package). Then, the probe name was converted into an international standard gene symbol. Gene differential expression analysis was performed using the limma package (Ritchie et al., 2015). Genes with a corrected *p* < 0.05 and log2 fold change (FC) > 1 were considered to be DEGs. The integration of microarray data was performed by the “aggregateRanks” function (Kolde et al., 2012) following the Robust Rank Aggregation (RRA) procedure in R (Kolde et al., 2012).

### Protein-Protein Interaction Network Integration

Protein-protein interaction (PPI) network analyses were performed using the STRING database.^3^

### Gene Ontology Enrichment Analysis

Gene ontology (GO) functional enrichment analysis was performed using the PANTHER classification system in the gene ontology resource.^4^

### Bacterial Strains and Growth Conditions

In this study, we used a methicillin-susceptible *S. aureus* (MSSA) ST1792 strain which was isolated from a prosthetic joint infection (PJI) prosthesis (Tan et al., 2019). *S. aureus* samples were divided into three groups: The logarithmic growth phase group, the stationary growth group, and the biofilm group. For the logarithmic phase group, ST1792 was cultured in TSB at 37°C with shaking at 200 rpm for 3 h to enter the logarithmic phase. The samples were then treated with vancomycin, ciprofloxacin, and linezolid at the sub-minimum inhibitory concentration (MIC) for another 3 h. The stationary phase group was cultured in TSB and incubated for 6 h (37°C, at 200 rpm) to reach the stationary phase. The antimicrobials at sub-MICs were then applied to the samples for a further 3 h. In the biofilm group, *S. aureus* was first cultured for 12 h in six-well plates at 37°C in TSB supplemented with 0.25% glucose (TSBG) and then cultured for another 12 h with sub-MIC antimicrobials.

### Bacterial Minimum Inhibitory Concentration Assay

A macrodilution method was used to determine the MICs of different antimicrobials for *S. aureus* ST1792. Here, 100 μl of twofold serial dilutions of antimicrobials dissolved in TSB were added to 100 μl of TSB, which contained approximately 10^6^ CFU/ml, in a 96-well microplate. After overnight culture at 37^°^C, the optical densities at 600 nm (OD_600_) of the cells in the 96-well microplate were measured. Relatively high OD_600_ values were indicative of bacterial growth, while low OD_600_ values indicated no growth. The MICs of the antimicrobials were considered to be the lowest concentrations that suppressed growth. Linezolid, ciprofloxacin, and vancomycin were purchased from Aladdin (Shanghai, China). Linezolid and ciprofloxacin were dissolved in methanol and acetic acid, respectively, and were diluted in TSB.

### Growth Curve Assay

One single colony of ST1792 was isolated and cultured in TSB on a gyratory shaker (200 rpm) at 37°C. The ST1792 was cultured on a gyratory shaker (200 rpm) at 37°C overnight. The next day, ST1792 was diluted at a ratio of 1:100 and incubated in TSB medium on a gyratory shaker (200 rpm) at 37°C. OD_600_ values were recorded at intervals of 1 h for up to 12 h until the stationary phase was reached.

### *Staphylococcus aureus* RNA Isolation and Quantitative PCR

We used qPCR to verify our bioinformatic data. Cells from the three groups were harvested and disrupted by a tissue lyser (70 Hz, 30 s). Next, we used the RNeasy kit (EZBioscience, United States) to isolate RNA according to the manufacturer’s instructions. The quality and quantity of the RNA were measured using a Nanodrop spectrophotometer (Thermo Fisher Scientific, United States). After adding gDNA remover, 1 μg RNA was reverse-transcribed into cDNA using a RT-PCR kit (EZBioscience, United States). The cDNA was then used as the DNA template for qPCR, performed with two SYBR Green qPCR Master Mix kits (EZBioscience). We used the following thermal cycler conditions for DNA amplification: initial denaturation at 95°C for 5 min, followed by 40 amplification cycles at 95°C for 10 s and 60°C for 30 s, using a Roche LightCycler 480 (Roche, Switzerland). Relative gene expression levels were quantified using the 2^–ΔΔ*CT*^ method (Yu et al., 2021), and *gyrB* expression levels were used as the internal reference. Each group and each selected gene had three replicates. The premier information is available in Supplementary Table 1.

## Results

### The Information on Microarray Data

**FIGURE 1:**
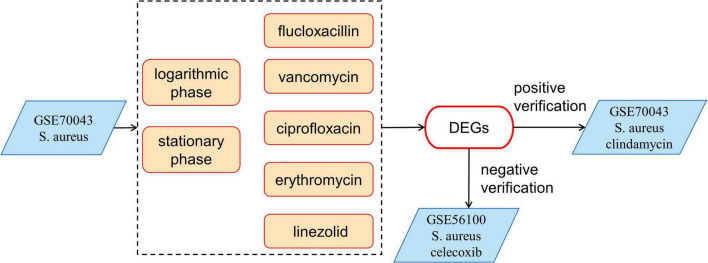
The bioinformatics processes used in this study. The expression data of GSE70043 was used as modeling data. GSE56100 was used as a negative control and the clindamycin treated samples were used as positive controls.

**FIGURE 2:**
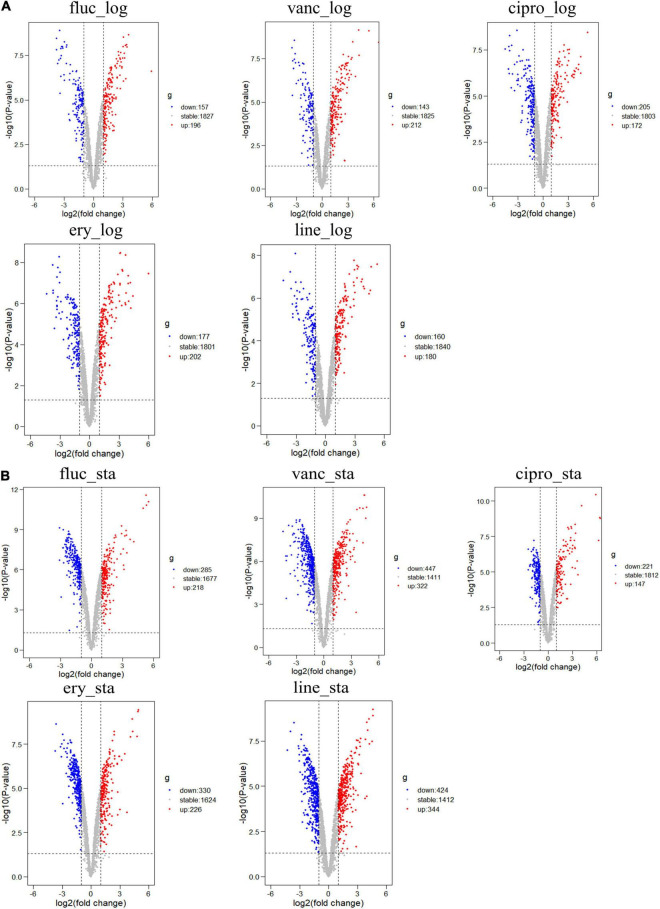
Differential expression of five different antimicrobials treated samples in logarithmic phase and stationary phase. **(A)** DEGs in logarithmic phase **(B)** DEGs in stationary phase. The red points represent upregulated genes screened based on log2 fold change > 1 and a corrected *p* < 0.05. The blue points represent the downregulation of the expression of genes screened based on log2 fold change < –1 and a corrected *p* < 0.05. The gray points represent genes with no significant differences.

The GSE70043 data set contains transcriptional data of NCTC 8325, a derivative strain of *S. aureus* (Herbert et al., 2010). The antimicrobials treated samples of the dataset were analyzed in depth in our study. *S. aureus* was grown in the presence of different antimicrobials including flucloxacillin (fluc), vancomycin (vanc), ciprofloxacin (cipro), clindamycin (clind), erythromycin (ery), and linezolid (line) and the samples were collected during the logarithmic and stationary phases. The GSE56100 contains transcriptional data of *S. aureus* ATCC29213, which was treated with celecoxib. GSE56100 was used as the negative control in the study to confirm that the DEGs identified in GSE70043 were specific for the antimicrobials. The bioinformatic processes used in this study are shown in Figure 1. The GSE70043 data set was screened using the limma package in R (corrected *p* < 0.05, |log2 FC| > 1) to identify DEGs (Figure 2).

### Identification of Differentially Expressed Genes Using Integrated Bioinformatics

**FIGURE 3:**
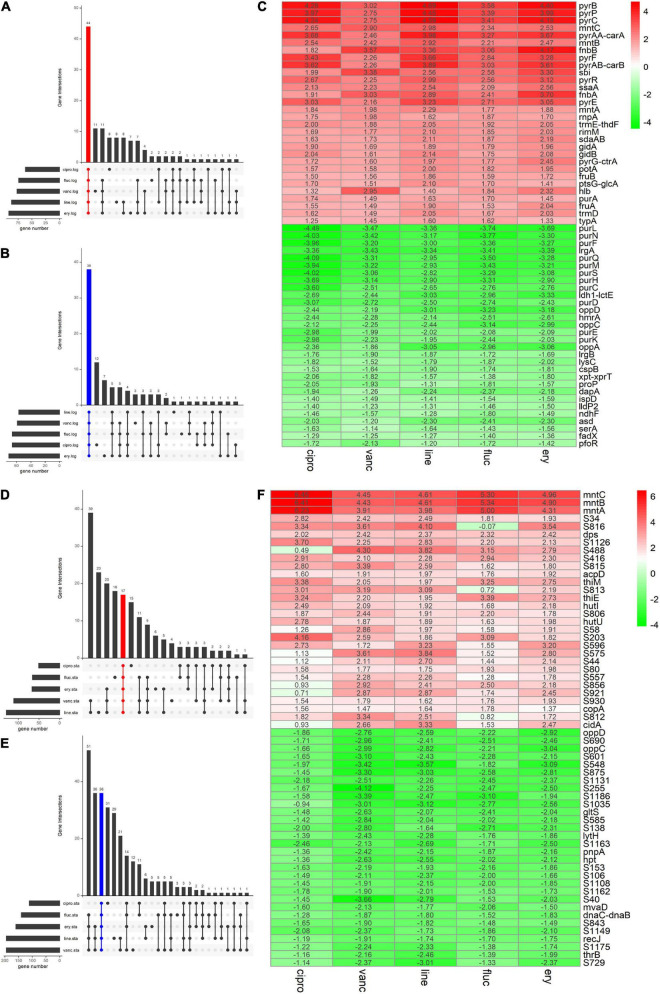
The Venn diagram and the Heatmap of the integrated DEGs **(A,B)** number of integrated up- and downregulated genes in the logarithmic phase are shown in a Venn diagram. **(D,E)** Number of integrated up- and downregulated genes of stationary phase shown in a Venn diagram. **(C)** Heatmap of the top 30 up- and downregulated genes in logarithmic phase. **(F)** Heatmap of the top 30 up- and downregulated genes in stationary phase. The abscissa is the antimicrobials, and the ordinate is the gene name. Red represents log2 FC > 0, green represents log2 FC < 0, and the values in the box represent the log2 FC values.

**FIGURE 4:**
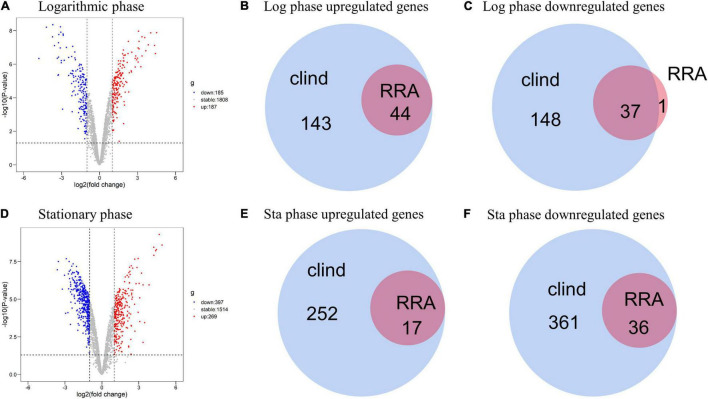
Volcano plots of DEGs for clindamycin treated samples and Venn diagrams of DEGs identified in clindamycin treated samples and integrated data. **(A)** Volcano plots of DEGs identified in clindamycin treated samples during the logarithmic phase. **(B,C)** Venn diagrams of DEGs identified in clindamycin treated samples and integrated data during the logarithmic phase. **(D)** Volcano plots of DEGs identified in clindamycin treated samples during stationary phase. **(E,F)** Venn diagrams of DEGs identified in clindamycin treated samples and integrated data during stationary phase.

We adopted the RRA method and through rank analysis, we identified 82 DEGs in the logarithmic phase including 44 upregulated and 38 downregulated genes (Figures 3A–C and Table 2). We also identified 53 DEGs in the stationary phase. Among them, 17 up- and 36 downregulated genes were obtained (Figures 3D–F and Table 3). Unannotated DEGs were excluded. A heat map of the top 30 up-and downregulated genes was generated, as shown in Figure 3. The clindamycin treated samples were used as positive controls, 372 DEGs were identified in the logarithmic phase including 187 up- and 185 downregulated genes; 666 DEGs were identified in the stationary phase. Among which, 269 up- and 397 downregulated genes were found (Figures 4A,D). The DEGs identified in the clindamycin treated samples included all of the genes that were differentially expressed in the integrated data, except *tatC* which was downregulated during the logarithmic phase in the integrated data but not downregulated in clindamycin treated samples (Figures 4B,C,E,F). For the GSE56100 data set, the celecoxib treated samples, 12 up- and 16 downregulated genes were found. None of these overlapped with the DEGs identified in the integrated data.

**TABLE 2:** Screening DEGs in logarithmic phase by integrated microarray.

DEGs	Gene names
Upregulated	ktrA potA fnbA rpmH nuc2 rplS pyrB pyrR chp gidB mntB rpmE2 rim pyrE trmD fruA glcA pyrP gidA fruB potC ssaA sbi potB carA fnbB sdaAB mntA hlb-1 carB potD truB pyrG plsX rplY rnpA purA pyrC trmE mntC hprT pyrF typA efb
Downregulated	gmk lctP2 purL purH purK ldh1 purQ purD pycA purF purM purE xpt purS cspB purN lrgB trpC opp-3D opp-3A norA opp-4C isaB purC serA guaA tarI fadX lysC dapA mpsA proP hmrA lrgA tatC metF asd pfoR

*DEGs, differentially expressed genes.*

**TABLE 3:** Screening DEGs in stationary phase by integrated microarray.

DEGs	Gene names
Upregulated	yidC htsA hutU mntA copA saHPF perR dps frp azoR mntB hutI isdI sbi thiE thiM mntC
Downregulated	pnpA isaA opp-3D dapD opp-3A tyrS atpC thrB gltS hsdS1 rplO dnaB relA tgt ilvH ilvB opp-4C atpD ilvD opuD1 thrC acpP mvaK1 lytH pycA dapA mvaD dapB dtd atpG cspB dnaC recJ asd hprT rpsG

*DEGs, differentially expressed genes.*

### Analyzing Differentially Expressed Genes Using a Protein-Protein Interaction Network Analysis and Gene Ontology Enrichment

**FIGURE 5:**
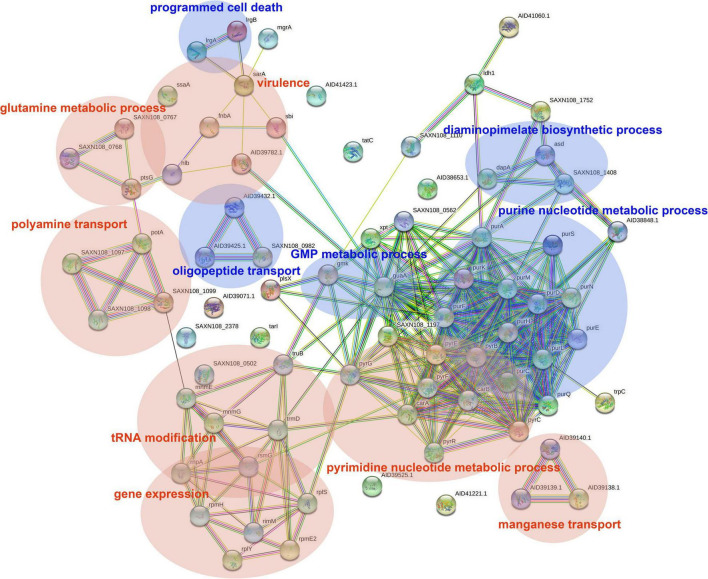
PPI network of DEGs in logarithmic phase. PPI, protein–protein interaction. Circles represent genes, lines represent protein interactions, and protein structures are shown in circles. Line color represents evidence of the interaction between the proteins.

**FIGURE 6:**
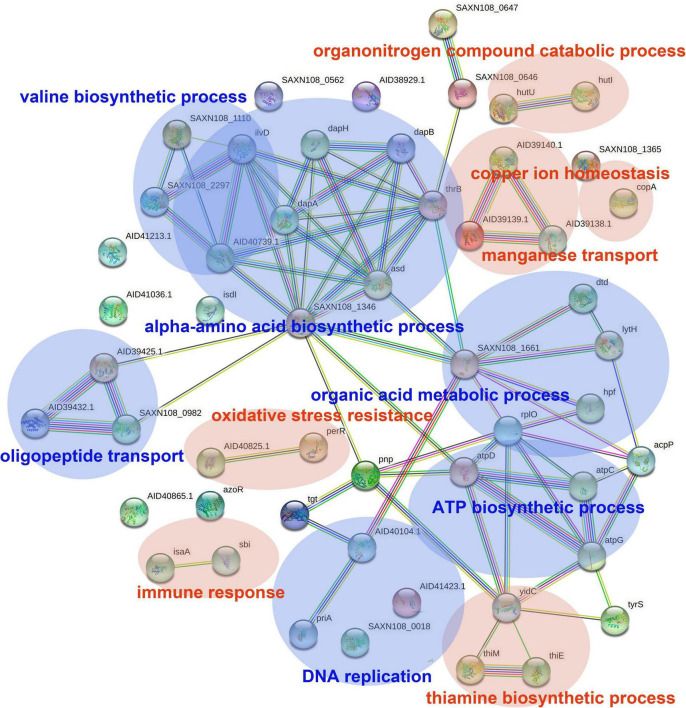
PPI network of DEGs in stationary phase. PPI, protein–protein interaction. Circles represent genes, lines represent protein interactions, and the structures of proteins are shown in the circles. Line color represents evidence of the interaction between the proteins.

The DEGs in antimicrobial-treated *S. aureus* were constructed into PPI networks using the STRING database (see text footnote 3). Complex networks of DEGs during the logarithmic and stationary phases were also constructed (Figures 5, 6). The 18 most significant genes showing strong interactions in the logarithmic phase were *guaA, purA, purH, carA-B, purK-N, pyrB-C, purQ, purC-F, hprT, pyrP.* In addition, 13 node genes with significant interactions in the stationary phase were identified, *thrC, relA, thrB, asd, rplO, ilvD, yidC, ilvH, atpD, atpG, dapA, acpP, dapB.*

**FIGURE 7:**
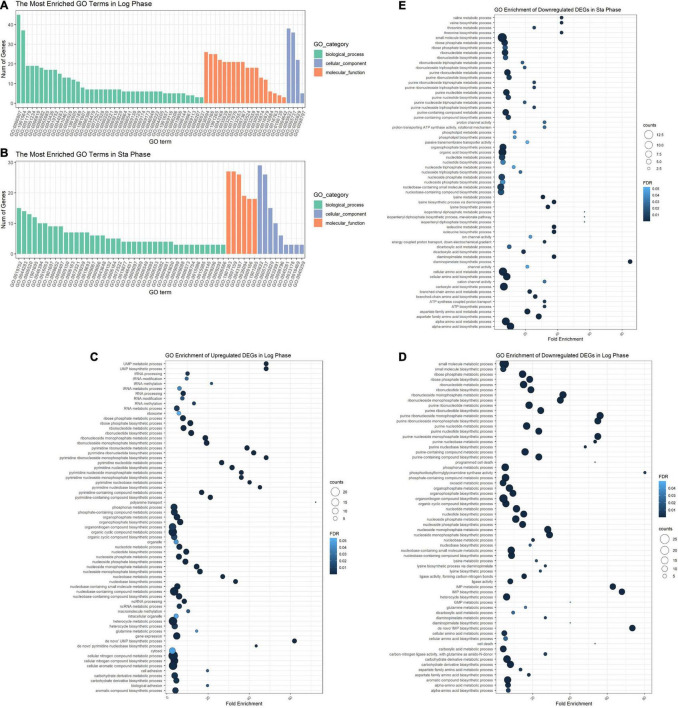
GO enrichment analysis of DEGs. GO analysis divided DEGs of the logarithmic phase **(A)** and stationary phase **(B)** into three functional groups: biological processes, molecular function, and cellular component. GO enrichment significance items of upregulated **(C)** and downregulated genes **(D)** during the logarithmic phase, and downregulated genes during the stationary phase **(E)**. DEGs, differentially expressed genes; GO, gene ontology.

The DEGs in the logarithmic phase were mainly enriched in transmembrane transport, “*de novo*” IMP/UMP biosynthesis, glutamine metabolism, and pyrimidine nucleotide metabolism/biosynthesis (Figure 7A). The DEGs identified in the stationary phase were mainly involved in energy coupled proton transport, down electrochemical gradient, threonine biosynthetic process, valine biosynthetic processes, diaminopimelate biosynthetic processes, and purine nucleotide metabolic/biosynthetic processes (Figure 7B). For the DEGs identified during the logarithmic phase, the upregulated genes were significantly enriched in polyamine transport, “*de novo*” UMP biosynthesis, UMP metabolism/biosynthesis, tRNA methylation, and cell adhesion (Figure 7C). The identified downregulated genes during the logarithmic phase were mainly involved in programed cell death, “*de novo*” IMP biosynthesis, IMP metabolism/biosynthesis, and purine ribonucleoside monophosphate metabolism/biosynthesis (Figure 7D). Regarding the downregulated genes during the stationary phase, these DEGs were enriched in diaminopimelate biosynthetic processes, isopentenyl diphosphate/biosynthetic and metabolic process, ATP biosynthesis, and cation channel activity (Figure 7E).

### qPCR Verification

**FIGURE 8:**
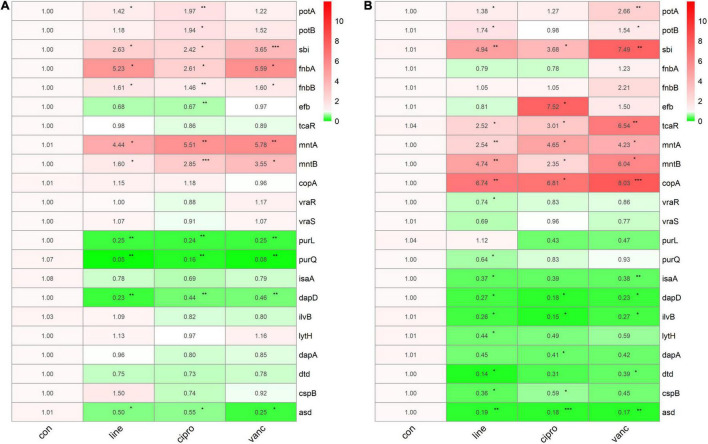
The qPCR data of antimicrobials treated ST1792. The gyrB normalized transcription of ST1792 during logarithmic phase **(A)** and stationary phase **(B)**. Statistical significance was calculated using ANOVA with Dunnett multiple column comparisons. *p < 0.05; ^**^p < 0.01; ^***^p < 0.001 vs. non-treated control.

We performed qPCR to verify our bioinformatic data against 22 genes and three antimicrobial agents (linezolid, vancomycin, and ciprofloxacin). The three antimicrobials were added into the medium at sub-MIC levels during the logarithmic and stationary growth phases of the ST1792 MIC and sub-MIC for strain ST1792 are shown in Table 4. Cell samples were collected after 3 h of treatment. The qPCR data showing transcriptional changes (Figure 8) confirm our bioinformatics data. Upon exposure to antimicrobial drugs, *S. aureus* significantly upregulates the expression of *fnbB, sbi, fnbA, mntA*, and *mntB*, and downregulates the expression of *purL, purQ, dapD*, and *asd* during the logarithmic phase (Figure 8A). While *sbi, tcaR, mntA, mntB*, and *copA* were significantly upregulated, *dapD, ilvB*, and *asd* were downregulated during the stationary phase (Figure 8B).

**TABLE 4:** Minimum inhibitory concentration (MIC) and sub-MIC for strain ST1792.

ST1792	Ciprofloxacin	Linezolid	Vancomycin
MIC (μg/mL)	0.25	1	2
Sub-MIC (μg/mL)	0.125	0.5	1

### qPCR Data for Virulence and Metabolism Adaptive Changes in *Staphylococcus aureus* Biofilms

**FIGURE 9:**
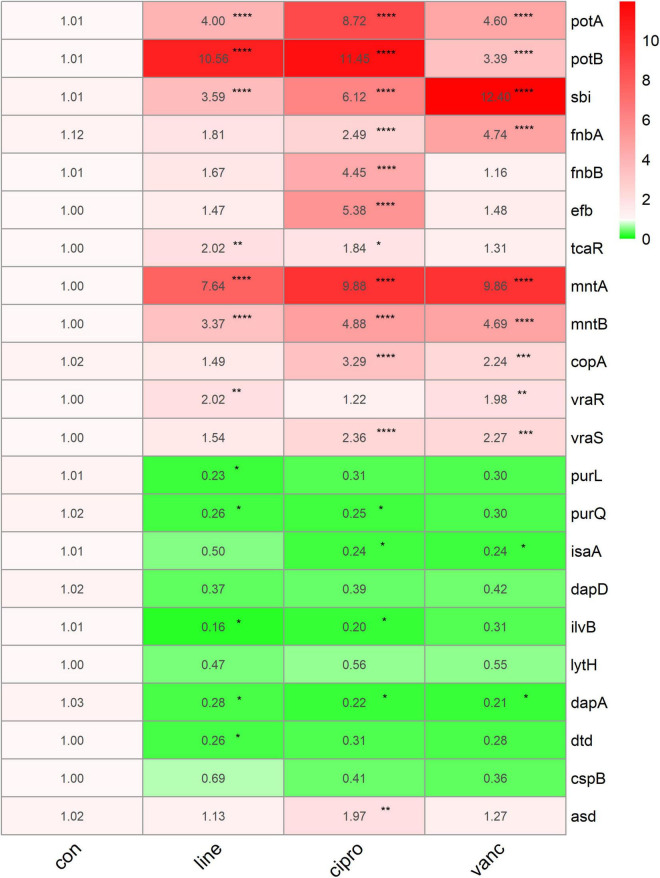
The qPCR data of antimicrobials treated ST1792. The gyrB normalized transcription after biofilm formation. Statistical significance was calculated using ANOVA with Dunnett multiple column comparisons. *p < 0.05; ^**^p < 0.01; ^***^p < 0.001; ^****^p < 0.0001 vs. non-treated control.

To further investigate the adaptive transcriptional changes in *S. aureus* biofilms when exposed to antimicrobials, we used qPCR to measure the 22 selected genes identified in the microarray datasets. The following genes were found to be upregulated: *potA, potB, sbi, fnbA, mntA, mntB, vraR, vraS, copA, fnbB, efb, asd*, and *tcaR*. In comparison, the following were downregulated: *purl*, *dapA, purQ, isaA, ilvB*, and *dtd*. Moreover, *potA, potB, sbi, mntA*, and *mntB* were significantly upregulated when exposed to linezolid, ciprofloxacin, and vancomycin, whereas *dapA* was downregulated. Ciprofloxacin and vancomycin increased *fnbA, copA*, and *vraS* expression, but decreased *isaA* expression. In addition, the expression of *vraR* increased in response to vancomycin and linezolid. For *purQ*, and *ilvB* expression, downregulation was observed after exposure to linezolid and ciprofloxacin, which also resulted in an increase in the *tcaR* expression. Downregulated *purL* and *dtd* were only identified in linezolid treated cells while *fnbB, efb*, and *asd* were upregulated following ciprofloxacin treatment (Figure 9).

## Discussion

Biofilm formation of *S. aureus* is the major cause of IAIs. Antimicrobial drugs are effective treatments against *S. aureus* infections (Hodille et al., 2017), with high doses being able to eliminate bacteria. However, adaptive changes may occur in *S. aureus* due to extreme selection pressure, which can result in antimicrobial resistance. Our study of the adaptive transcriptomic changes in *S. aureus* biofilm formation, virulence, and metabolism following exposure to different antimicrobial agents, especially in the common transcriptional features, is required to improve our understanding of the molecular mechanisms behind proliferation and antimicrobial resistance of *S. aureus.* This has clinical significance for the prevention of *S. aureus*-induced IAIs.

Using a combination of publicly available bioinformatic data and qPCR experiments, we found that *mntA-C* and *sbi* were significantly upregulated in the logarithmic phase, stationary phase, and during biofilm formation. Interestingly, *mntA-C* encodes genes involved in the manganese transport system ATP-binding proteins. Previous studies have shown that manganese plays an important role in activating or stimulating enzymes involved in stress responses, central carbon, and general metabolism in *Streptococcus pneumoniae* (Ogunniyi et al., 2010). For *S. aureus*, MntABC is essential for maintaining resistance to oxidative stress and superoxide dismutase (SOD) activity (Handke et al., 2018). A lack of manganese ions can cause disorders in metabolic processes and increase vulnerability to the host defense system (Radin et al., 2018). In this study, we found that following treatment with different antimicrobials, *S. aureus* specifically upregulated *mntA-C* expression. This demonstrates the importance of manganese in antibiotic stress responses and in the stability of the metabolic process.

The *sbi* gene encodes the IgG-binding protein Sbi, which plays an important role in the inhibition of both the innate and adaptive immune responses. Sbi possesses two N-terminal domains that bind the Fc region of IgG and two domains that form a tripartite complex with complement factors C3b and CFH. By recruiting CFH and C3b (Koch et al., 2012), the secreted form of Sbi acts as a potent complement inhibitor of alternative pathway-mediated lysis (Haupt et al., 2008). The increased transcriptional expression of *sbi* may be related to the immune evasion of *S. aureus* under antimicrobial selection pressure.

We also identified downregulation of *opp-3D, opp-4C, opp-3A, cspB*, and *dapA* in both logarithmic and stationary growth phases. Further, *opp-3D, opp-4C*, and *opp-3A* produce the oligopeptide transport system ATP-binding proteins. Decreased transcriptional expression of the oligopeptide transport system may be associated with the defense of exogenous proteins. Dihydrodipicolinate synthase, the product of *dapA*, is an essential enzyme involved in the lysine biosynthesis pathway (Girish et al., 2008). Cold shock protein B (CspB), encoded by *cspB*, responds to cold stress (Catalan-Moreno et al., 2021). A recent study revealed the production of *S. aureus* CspB and CspC is controlled by two paralogous RNA thermoswitches which repress CspB and CspC production at 37°C (Catalan-Moreno et al., 2021). Upon exposure to high temperature, the downregulated *cspB* in *S. aureus* may decrease susceptibility to daptomycin, gentamicin, and trimethoprim-sulfamethoxazole (TMS) (Duval et al., 2010). These down-regulated genes may play a crucial role in antimicrobials resistance.

During the logarithmic growth phase, *S. aureus* significantly upregulated the *potA-D* gene, which encodes spermidine/putrescine import ATP-binding proteins. We consider this to be an antimicrobial-induced adaptive transcriptional change in *S. aureus*. Spermidine and putrescine are polyamines and are thought to help stabilize some membranes and nucleic acid structures (Kwon and Lu, 2007). However, for *S. aureus*, polyamines are not only irrelevant but are detrimental (Li et al., 2019). *S. aureus* has lost most polyamine biosynthetic genes to overcome the toxicity of high levels of polyamines (Yao and Lu, 2014). The mechanisms behind some antimicrobials, such as clindamycin, erythromycin, and linezolid, involve disruption of protein synthesis. The observed upregulation of PotA-D may increase spermidine/putrescine levels in *S. aureus* to promote stability during protein synthesis. We found that *potA-D* was also upregulated in the biofilm stage. The increased transcriptional expression of *trmD* is associated with tRNA modification which is also an important adaptive change that stabilizes protein synthesis (Zhang et al., 2017).

*lrgA* and *lrgB* are involved in the negative control of murein hydrolase activity and autolysis (Groicher et al., 2000). Therefore, decreased expression of *lrgA* and *lrgB* can increase murein hydrolase activity. During the stationary phase of *S. aureus*, reduced expression of LrgA and LrgB enhance penicillin-induced killing of cells (Groicher et al., 2000), which is disadvantageous for *S. aureus* survival. However, here we observed downregulation of *lrgA* and *lrgB* during the logarithmic phase, a time in which *lrgAB* expression was shown to be minimal (Groicher et al., 2000). It is well established that peptidoglycan hydrolases are necessary to cleave the septum to allow the separation of daughter cells (Vollmer et al., 2008). Previous research has shown that a specific lrgAB mutant is capable of increasing levels of eDNA in the biofilm matrix, resulting in a more adherent biofilm (Sharma-Kuinkel et al., 2009). Therefore, decreased expression of *lrgA* and *lrgB* could be beneficial to the proliferation of *S. aureus* and biofilm formation during the logarithmic phase. In addition, we observed increased expression of adhesion genes *efb, fnbA*, and *fnbB*, which may also promote biofilm formation in *S. aureus* (Foster et al., 2020).

Genes involved in purine metabolism (*purl, purH, purK, purQ, purD, purF, purM, purE, purS, and purN*) were downregulated, while those involved in pyrimidine metabolism (*pyrB, pyrR, pyrE, pyrP, carA, pyrG, pyrC, and pyrF*) were upregulated in the logarithmic phase. Inactivation of the *S. aureus* purine biosynthesis repressor leads to hypervirulence (Goncheva et al., 2019). Downregulated purine metabolism leads to reduced virulence which may be a mechanism of survival for *S. aureus*. Pyrimidine synthesis was significantly upregulated following antimicrobials treatment, which enhanced the resistance of *S. aureus* to reactive oxygen species (Buvelot et al., 2021). During the stationary growth phase, the expression of genes involved in amino acid biosynthesis, ATP biosynthesis (*atpC, atpD, atpG*), and DNA replication (*dnaB, dnaC*) were downregulated. These transcriptional changes may be associated with bacterial dormancy and may lead to persisters, a phenotypic transformation that generates bacteria with reduced sensitivity to antibiotics (Conlon et al., 2016). According to our qPCR data (Figure 9) of the biofilm stage, the transcriptomic changes seem to be more significant than in the logarithmic and stationary phases. In the biofilm stage, *S. aureus* upregulated genes involved in the polyamine transport system, manganese transport system, and adhesion genes which can stabilize protein synthesis, and metabolic processes, and reinforce the biofilm structure. These adaptive transcriptional expressions may be associated with biofilm resistance to antimicrobials.

*S. aureus* utilizes many survival strategies, either by defending against external stress or adapting to environmental conditions. *S. aureus* can survive the host immune response owing to the expression of a wide range of virulence factors that interfere with the host immune defenses (Watkins and Unnikrishnan, 2020). Biofilm formation can enhance resistance toward antimicrobials as well as resistance to immune response. In response to external pressure, actively growing *S. aureus* can switch lifestyle to promote biofilm formation, change its metabolism, surface properties, and produce a highly protective EPS. The biofilm includes the quasi-dormant and potentially persistent cells that are tolerant to antibiotics (Bui et al., 2017). In recent years, it has become clear that *S. aureus* is able to invade and survive in a range of cell types. The ability to survive intracellularly provides *S. aureus* with another way to evade antibiotics and immune responses during infection (Goldmann and Medina, 2018).

The DEGs identified in this study following exposure of cells to antibiotic stress have important implications for the survival of *S. aureus*. For example, we observed upregulation of the *sbi* gene, which plays a role in the inhibition of both the innate and adaptive immune responses. The expression of adhesion genes *efb, fnbA*, and *fnbB* was upregulated, which may promote *S. aureus* biofilm formation (Foster et al., 2020). The ATP biosynthetic process (*atpC, atpD*, and *atpG*), and DNA replication (*dnaB* and *dnaC*) were downregulated during the stationary growth phase. Recent studies have confirmed cellular ATP depletion by persister cells as a key feature and the basis for their tolerance to a range of antibiotics (Conlon et al., 2016).

The GSE70043 is the main dataset used in our study. The previous study (Mäder et al., 2016) has performed the analysis of all RNA transcripts of *S. aureus* under various conditions ranging from *in vitro* growth in different media to internalization by eukaryotic host cells, in order to identify factors regulating the transcriptome architecture of *S. aureus*. However, the transcriptome characteristics of *S. aureus* under antibiotic stress have not been explored in-depth in the previous study. Therefore, the integrated analysis of the antibiotics treated samples in the dataset was performed in our study to identify the common adaptive transcriptional changes induced by antibiotic stress.

The previous study (Mäder et al., 2016) added sub-MIC of the antimicrobials at the beginning of the cultures and collected the samples at the logarithmic growth phase as well as 4 h after entry into the stationary phase for the microarray. Our study used a different intervention way in the qPCR verification which is mentioned in the methods part. We treated the *S. aureus* with antimicrobials at sub-MIC for 3 h after the bacteria enter the logarithmic and stationary growth phase, respectively. Although the sub-MIC antibiotics could not inhibit the growth of *S. aureus*, they do impact the growth curve of bacteria (Weiss et al., 2016). The intervention method used in qPCR could reduce the influence of antibiotics on the growth curve. What’s more, the unified intervention time in the logarithmic group and stationary group could avoid bias from the antibiotics treatment time.

There are several limitations to our study. First, we focused only on the transcriptomic changes in MSSA. The adaptive transcriptional expressions of MRSA under the impact of antimicrobials are also important. We actually performed qPCR experiments to investigate the transcriptional levels of USA300 (MRSA strain), however, the expression tendency in MRSA is different from that in MSSA. This suggests that the transcriptomic changes observed in MRSA upon exposure to antimicrobial agents require further study. Second, six antimicrobials were included in this study. The impact of other antimicrobial drugs, such as rifampin and daptomycin should also be investigated. Finally, there was no microarray or sequencing data of biofilms in the study. In the future, microarrays or RNAseq of antimicrobials-treated biofilms should be performed.

In summary, under the pressure of different antimicrobials, *S. aureus* specifically upregulated the manganese import system gene, *mntA-C*, and the immune response gene, *sbi*, in all studied stages to enhance resistance to oxidative stress and inhibit the host immune responses. During the logarithmic phase, the adaptive transcriptomic changes of *S. aureus* mainly involved the stability of protein synthesis, adhesion, and biofilm formation to promote growth and survival under the pressure of antimicrobial treatment. In the stationary phase, *S. aureus* significantly downregulated the expression of genes involved in ATP synthesis and DNA replication, which may lead to the *S. aureus* dormancy and tolerance to the antimicrobials.

Herein, we provide a general map of transcriptional expression of virulence, metabolism, and biofilm formation following adaptive changes in *S. aureus* after exposure to different antimicrobials. These data have important clinical significance for the prevention of *S. aureus* infections and they provide novel therapeutic targets against *S. aureus* infection.

## Data Availability Statement

Publicly available datasets were analyzed in this study. This data can be found here: The microarray data (GSE70043 and GSE56100) are available on the GEO DataSets database (https://www.ncbi.nlm.nih.gov/geo/).

## Author Contributions

ZR contributed to the concept of the study and wrote the manuscript. ZR and JY contributed to the public databases research. FJ and FZ contributed to the bioinformatics analyses. JD, BW, and YZ contributed to the qPCR experiment. JT and MH collected clinical strains and performed antibiotic susceptibility testing. HS contributed to the data analysis and data interpretation. PH contributed to the study design, manuscript editing, and revision. All authors contributed to the article and approved the submission of this manuscript.

## Conflict of Interest

The authors declare that the research was conducted in the absence of any commercial or financial relationships that could be construed as a potential conflict of interest.

## Publisher’s Note

All claims expressed in this article are solely those of the authors and do not necessarily represent those of their affiliated organizations, or those of the publisher, the editors and the reviewers. Any product that may be evaluated in this article, or claim that may be made by its manufacturer, is not guaranteed or endorsed by the publisher.

## Footnotes

1
https://www.ncbi.nlm.nih.gov/geo/query/acc.cgi?acc=GSE70043

2
https://www.ncbi.nlm.nih.gov/geo/query/acc.cgi?acc=GSE56100

3
http://string-db.org

4
http://geneontology.org/

## Supplementary Material

Supplementary Figure 1 Growth curve of the ST1792.

Supplementary Figure 2 PCR verification of the qPCR primer product size and primer efficiency.

Supplementary Figure 3 Standardization of gene expression GSE70043.

Supplementary Figure 4 Standardization of gene expression GSE56100.

Supplementary Table 1 Primer sequences for qPCR.
