# Supplementary figures and images for: A General Map of Transcriptional Expression of Virulence, Metabolism, and Biofilm Formation Adaptive Changes of Staphylococcus aureus When Exposed to Different Antimicrobials
Source: Front Microbiol. 2022 Jun 17;13:825041. doi: 10.3389/fmicb.2022.825041 (PMC9247510; doi:10.3389/fmicb.2022.825041)

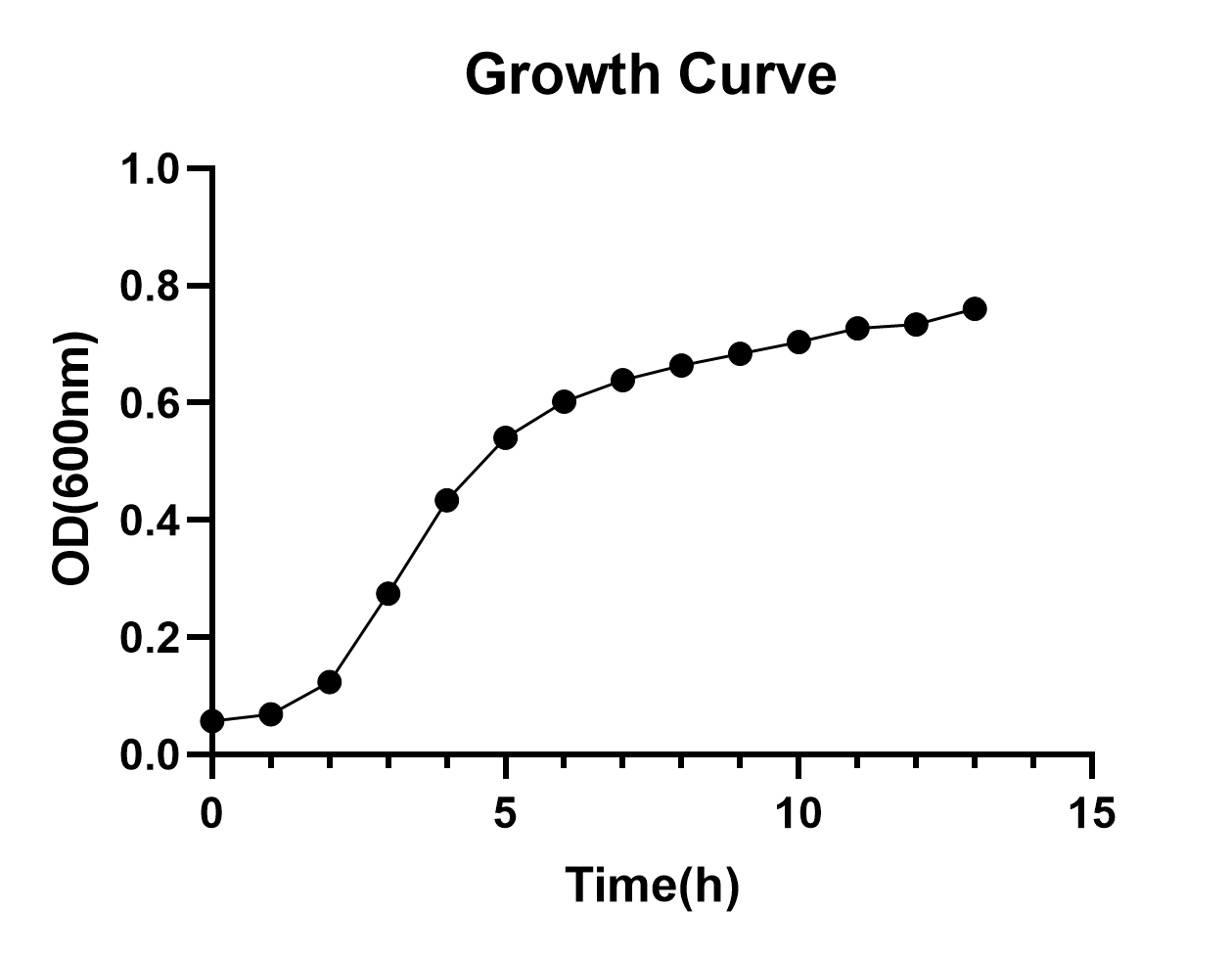

Supplement: Supplementary Figure 1 — Growth curve of the ST1792. [file Image_1.TIF]

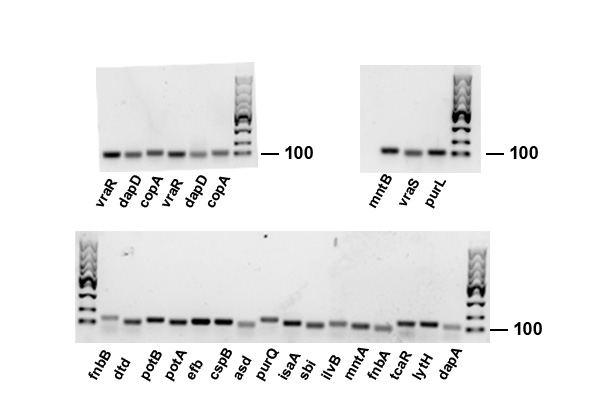

Supplement: Supplementary Figure 2 — PCR verification of the qPCR primer product size and primer efficiency. [file Image_2.JPEG]

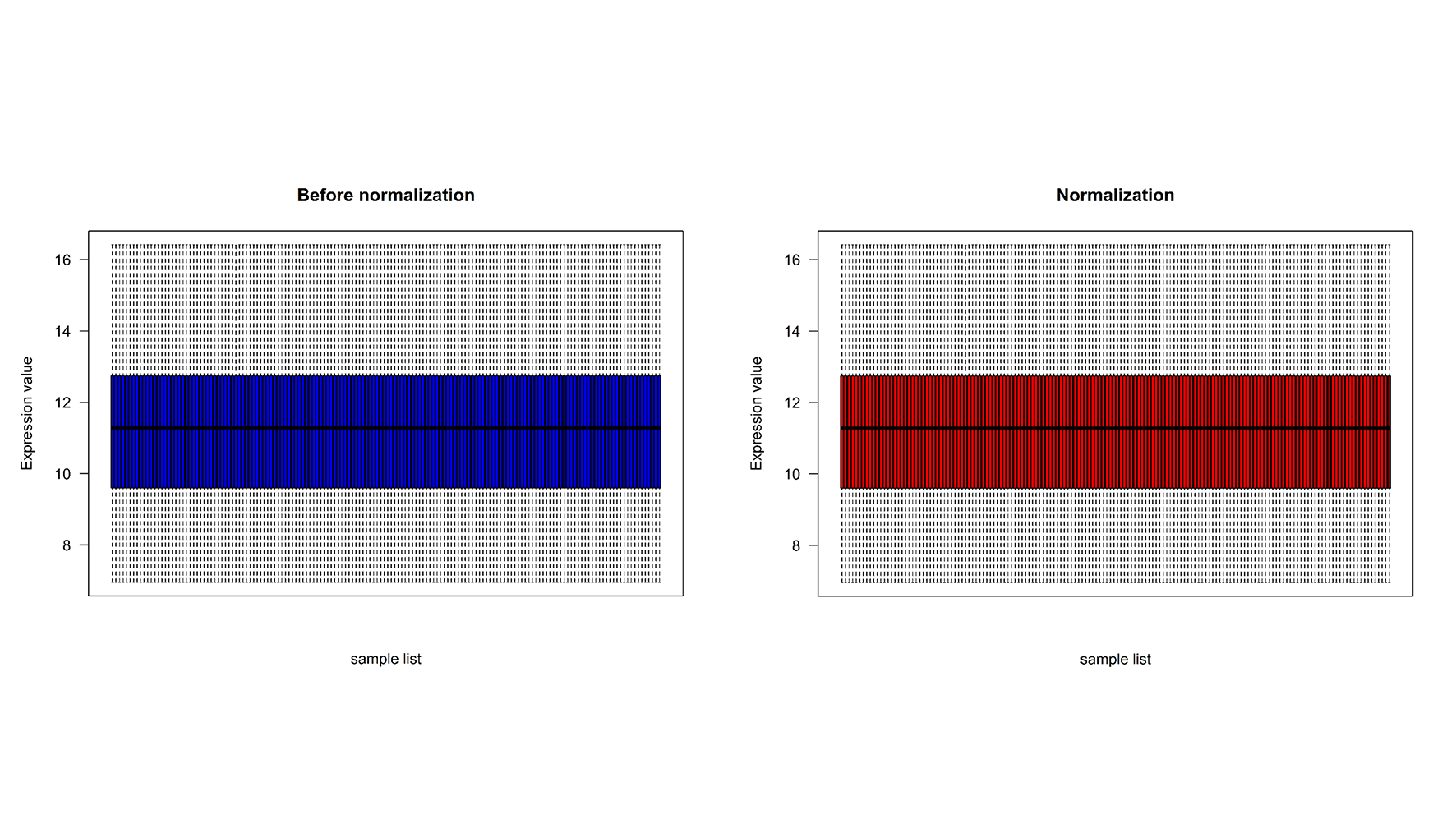

Supplement: Supplementary Figure 3 — Standardization of gene expression GSE70043. [file Image_3.TIF]

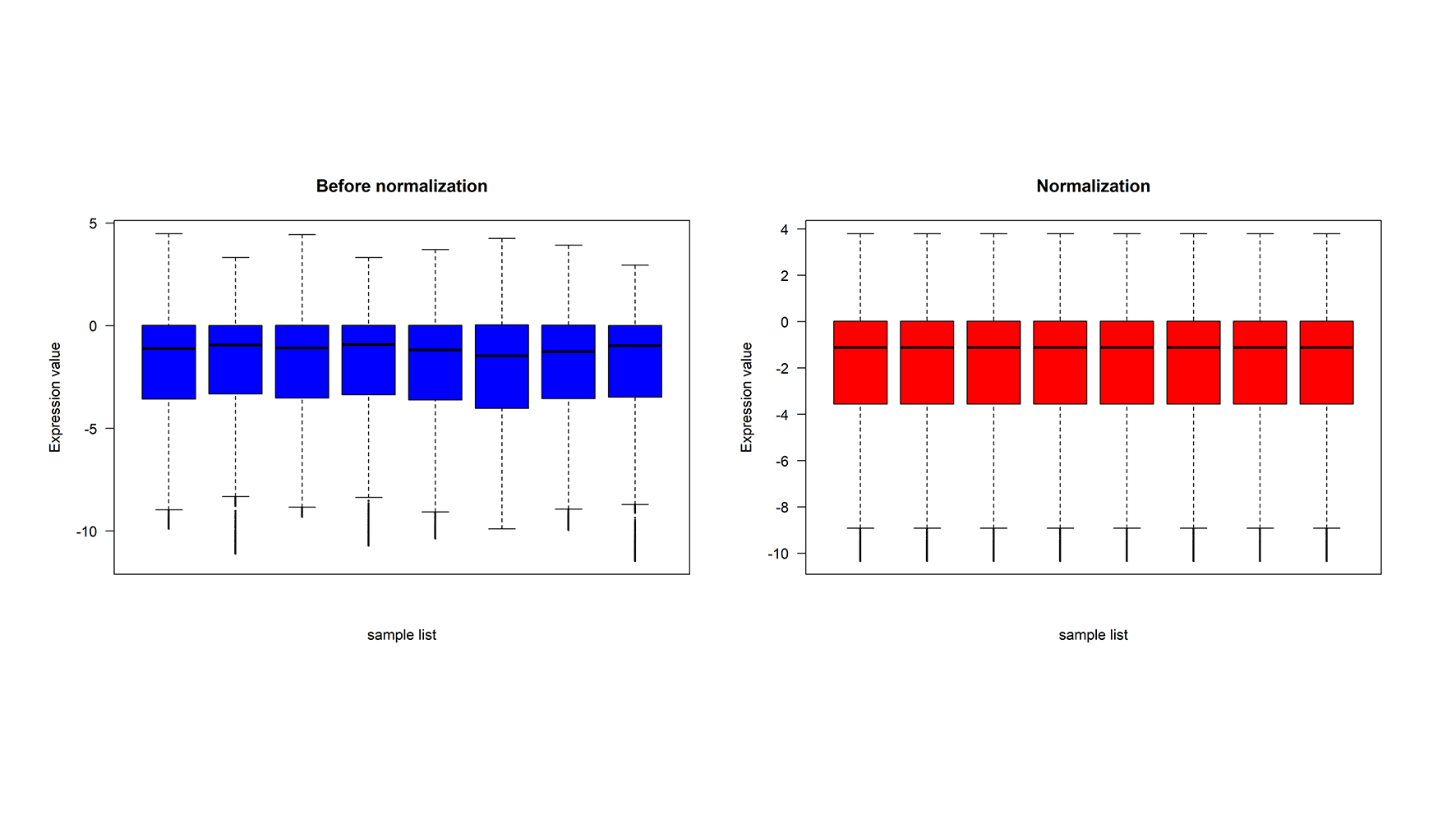

Supplement: Supplementary Figure 4 — Standardization of gene expression GSE56100. [file Image_4.TIF]
